# Identification, Genetic Analysis, and Pathogenicity of Classical Swine H1N1 and Human-Swine Reassortant H1N1 Influenza Viruses from Pigs in China

**DOI:** 10.3390/v12010055

**Published:** 2020-01-02

**Authors:** Yafen Song, Yong Zhang, Bing Zhang, Ling Chen, Min Zhang, Jingwen Wang, Ying Jiang, Chenghuai Yang, Taozhen Jiang

**Affiliations:** Department of Veterinary Culture Collection, China Institute of Veterinary Drug Control, 8 Nandajie, Zhongguancun, Haidian District, Beijing 100081, China; songyf86527@163.com (Y.S.); zhangyong_1537@163.com (Y.Z.); zhangbing06@163.com (B.Z.); chen2011521@163.com (L.C.); zmbooksea@163.com (M.Z.); echowongbj@gmail.com (J.W.); jiangying_vet@163.com (Y.J.); ychenghuai@163.com (C.Y.)

**Keywords:** swine influenza virus, H1N1, phylogenetic analysis, reassortant, molecular characterization, pathogenicity, mice

## Abstract

Swine influenza virus causes a substantial disease burden to swine populations worldwide and poses an imminent threat to the swine industry and humans. Given its importance, we characterized two swine influenza viruses isolated from Shandong, China. The homology and phylogenetic analyses showed that all eight gene segments of A/swine/Shandong/AV1522/2011(H1N1) were closely related to A/Maryland/12/1991(H1N1) circulating in North America. The HA, NA, M, and NS genes of the isolate were also confirmed to have a high homology to A/swine/Hubei/02/2008(H1N1) which appeared in China in 2008, and the virus was clustered into the classical swine lineage. The gene segments of A/swine/Shandong/AV1523/2011(H1N1) were highly homologous to the early human H1N1 and H2N2 influenza viruses, except for the HA gene, and the virus was a reassortant H1N1 virus containing genes from the classical swine (HA) and human (NA, PB2, PB1, PA, NP, M, and NS) lineages. Both the viruses could cause lethal infection and replicate efficiently in the lungs, brains, spleens, and kidneys of mice. Histopathological examinations showed that AV1522 and AV1523 viruses caused a spectrum of marked pneumonia and meningoencephalitis according to the duration of infection, demonstrating a progression of respiratory disease and neurological disease over the course of infection that ultimately resulted in lethality for the infected mice. The changes in the pathogenicity of swine influenza viruses to mammals, accompanied with the continuous reassortment and evolution of the viruses, highlights the importance of ongoing epidemiological investigation.

## 1. Introduction

Swine influenza virus (SIV) is a highly contagious viral infection of pigs that can cause significant economic impact on animal husbandry and poses a potential pandemic threat to humans. SIV is an enveloped virus with a segmented RNA genome, which belongs to the Influenza A viruses (IAV) of the family Orthomyxoviridae. The Influenza A viruses are further divided into subtypes based on their haemagglutinin (HA) and neuraminidase proteins (NA) [1]. There are currently 18 HA subtypes and 11 NA subtypes, most of which circulate in wild birds [2], and classical and avian H1N1, human H1N1 and H1N2, reassortant H3N2 (r), and rH1N2 are known to have circulated widely in pigs. Other subtypes, rH1N7, rH2N3, rH3N1, equine H3N8, H5N2, H6N6, H7N2, H4N8, H11N6, avian avH3N3, avH4N6, avH5N1, and avH9N2, have also been occasionally identified in pigs in different parts of the world [3,4,5,6,7,8,9,10].

Influenza A virus has remarkable ability in escaping host defense mechanisms by antigenic drift and antigenic shift [11]. Gradual changes of the antigenic properties are known as antigenic drift. Because of its polymerase complex lacking proofreading activity, numerous mutations accumulate in the viral genome during the ongoing replication cycle, which may result in a multitude of new variants being produced, allowing the new virus to rapidly adapt to changes in the environment. Due to the nature of its segmented genome, IAV is able to undergo reassortment when a host cell is concurrently infected with more than one virus, which is described as an antigenic shift [12].

Swine have played a central role in the ecology of influenza A virus and are widely known as a ‘‘mixing vessel’’. Swine appeared unique in having both α-2,3-SA and α-2,6-SA residues distributed throughout the respiratory tracts, whereas avian, swine, and human influenza viruses can undergo genetic reassortment resulting in the creation of novel viruses [13,14,15]. Once established in swine, the novel reassortment viruses would pose a substantial threat to swine populations and—with the introduction or reintroduction into the human population—would pose outbreak and pandemic risks. This risk is exemplified by the emergence of the pandemic H1N1 2009 virus—with gene segments of the virus derived from swine, human, and avian origin, which have circulated in swine populations in North America, Europe, Asia, and other regions for many years—which further emphasized the role of swine in the genesis of influenza reassortant variants with the potential to infect humans [16,17].

Therefore, understanding the evolution and pathogenicity of SIVs in China may help guide management and pandemic preparedness strategies. In this study, we phylogenetically characterized two SIV isolates obtained from pigs with respiratory disease in farms of Shandong, China. Additionally, to find out the virulence of the two viruses to mammals, the mouse mode was used to study the pathogenicity of two isolates.

## 2. Materials and Methods

### 2.1. Viruses Isolation and Identification

During a routine surveillance, two swine influenza viruses were isolated from the lung tissue samples collected from pigs with respiratory disease in two swine farms of Shandong, China in 2011. For virus isolation, the collected samples were homogenized in phosphate buffer saline (PBS) supplemented with a final concentration of 2000 units/mL penicillin and 2000 units/mL streptomycin and were centrifuged at 1000 g for 30 min at 4 °C to isolate supernatant fluids. The supernatant fluids were filtered through a filter of 0.45-μm pore size (Merck Millipore Ltd. Tullagreen Carrigtwohill Co. Cork, Ireland). Then, the filtered liquid was inoculated into the allantoic cavities of 9–10-day-old specific-pathogen-free (SPF) embryonated chicken eggs. After incubation at 37 °C for 72 h, the allantoic fluids were harvested. Haemagglutination (HA) assay with 1% chicken erythrocytes was performed to determine the presence of influenza virus. The subtype of the isolates was identified by polymerase chain reaction (RT-PCR) using subtype-specific primers [18,19].

### 2.2. Sequencing and Phylogenetic Analysis

RNA was extracted using TRIZOL LS Reagent (Invitrogen Life Technologies, Inc. Carlsbad, CA, USA) according to the standard protocol. Reverse transcription (RT) was carried out under standard conditions using the Uni12 (AGCAAAAGCAGG) primer. The PCR was performed using segment-specific primers for eight genes. The PCR products were purified with the QIAquick PCR purification kit (Qiagen, Hilden, Germany) and sequenced using an automatic ABI Prism 3730 genetic analyzer (Applied Biosystems) by Shanghai Invitrogen Biotechnology Co., Ltd. DNA sequences were compiled and edited using Lasergene 7.1 (DNASTAR, Madison, WI, USA). Phylogenetic trees were generated by the distance-based neighbor-joining method using software MEGA 4.0 (Sinauer Associates, Inc., Sunderland, MA, USA). The reliability of the tree was assessed by bootstrap analysis with 1000 replicates. Horizontal distances are proportional to genetic distance. The nucleotide sequences obtained in the present study are available from GenBank under the accession numbers: AV1522: MN700130, MN700108-MN700114; AV1523: MN700049-MN700056.

### 2.3. Mouse Experiments

To evaluate the pathogenicity of isolates in mammalian hosts, the six- to eight-week-old SPF female BALB/c mice were randomly divided into two groups of 11 mice each. The mice were inoculated intranasally with 10^6^ EID_50_ of each virus in a 50-μL volume after anesthesia with CO_2_. Additionally, 11 mice inoculated with 50 μL of PBS served as negative controls. Three mice were euthanized at 3 days post-infection (DPI) and 5 DPI, respectively. The lungs, brains, spleens, and kidneys collected from the infected and control mice were then taken for viral titer analysis in SPF embryonated chicken eggs by Reed and Muench. The remaining infected and control mice were monitored for weight loss and mortality for 14 days.

### 2.4. Histopathology

The six- to eight-week-old female BALB/c mice were inoculated intranasally with 50 μL (10^6^ EID_50_) of the AV1522 virus or AV1523 virus. Three mice from each group were euthanized at 3 DPI and 5 DPI. The lungs and brains of the inoculation mice were removed and fixed in 10% phosphate-buffered formalin. Specimens were then dehydrated and embedded in paraffin. Sections were cut to 5 μm and stained with hematoxylin and eosin (HE) for histopathologic examination.

### 2.5. Statistical Analysis

Statistical analyses were conducted using GraphPad Prism 5.0 software (GraphPad Software Inc., San Diego, CA, USA). Statistical analyses of the virus titer in the organs of control and infected mice were performed using a two-way ANOVA. The survival of mice was analyzed using log-rank (Mantel–Cox) tests and Gehan–Breslow–Wilcoxon tests. *p* values of <0.05 were considered significant.

### 2.6. Ethics Statement

The six- to eight-week-old SPF female BALB/c mice were purchased from Beijing Vital River Laboratory Animal Technology Co., Ltd., Beijing, China. The 9–10-day-old specific-pathogen-free (SPF) embryonated chicken eggs were purchased from Beijing Boehringer Ingelheim Vital Biotechnology Co., Ltd., China. The animal experiments in this study were performed in biosafety level 2+ facilities at the China Institute of Veterinary Drug Control (IVDC). The animal studies and embryonated chicken egg experiments were conducted in accordance with animal welfare guidelines and applicable laws and were approved by the Ethics Committee of IVDC (IVDCABSL201800061; 10 December 2018).

## 3. Results

### 3.1. Virus Isolation and Identification

Two viruses with hemagglutination activity were isolated from two lung samples of pigs with respiratory diseases from two farms in Shandong, China. The viruses were subsequently passaged three times with the inoculated of 9–10-day-old SPF embryonated chicken eggs by limiting dilution assay and were then tested. The two isolates were both identified and confirmed as H1N1 subtype influenza viruses by RT-PCR, genomic sequencing, and the nucleotide BLASTn analysis of the Influenza Sequence Datebase in GeneBank. The two viruses were designated as A/swine/Shandong/AV1522/2011(H1N1) (AV1522) and A/swine/Shandong/AV1523/2011(H1N1) (AV1523). Further genetic analysis and analyses of the biological characteristics of the two viruses were performed in this study.

### 3.2. Phylogenetic Analysis of H1N1 Viruses

To understand the genetic evolution of the two H1N1 viruses, the eight genes of each virus were sequenced. The eight phylogenetic trees were constructed using the nucleotide sequences of representative viruses available in the GenBank database.

The eight gene segments of the two H1N1 viruses isolated in 2011 were 83.3% to 99.5% homologous with each other, and they shared amino acid homologies ranging from 83.7% to 98.9%.

The eight gene segments of the AV1522 virus were closely related to A/Maryland/12/1991(H1N1) circulating in North America, with nucleotide sequence homologies ranging from 99.5% to 100%. The HA, NA, M, and NS genes of the isolate were also confirmed to have a high homology to A/swine/Hubei/02/2008(H1N1) which appeared in China in 2008, with nucleotide sequence homologies ranging from 99.5% to 100%. The HA gene of the AV1523 virus was closely related to A/Maryland/12/1991(H1N1), with a nucleotide sequence homology of 100%. The NA, PB2, PB1, and NS genes had the highest homologies to the early human H1N1 influenza virus (A/Alaska/1935(H1N1), with nucleotide sequence homologies ranging from 99.4% to 100%. The PA gene had 99.9% nucleotide sequence homology compared with A/Victoria/36/1988(H1N1). The NP gene shared the highest nucleotide sequence homology to the human H2N2 influenza virus (A/Ann Arbor/6/1960), with 99.7% identity. The M gene of the AV1523 virus had 99.8% nucleotide sequence homology compared with A/New Jersey/1976(H1N1).

**Figure 1:**
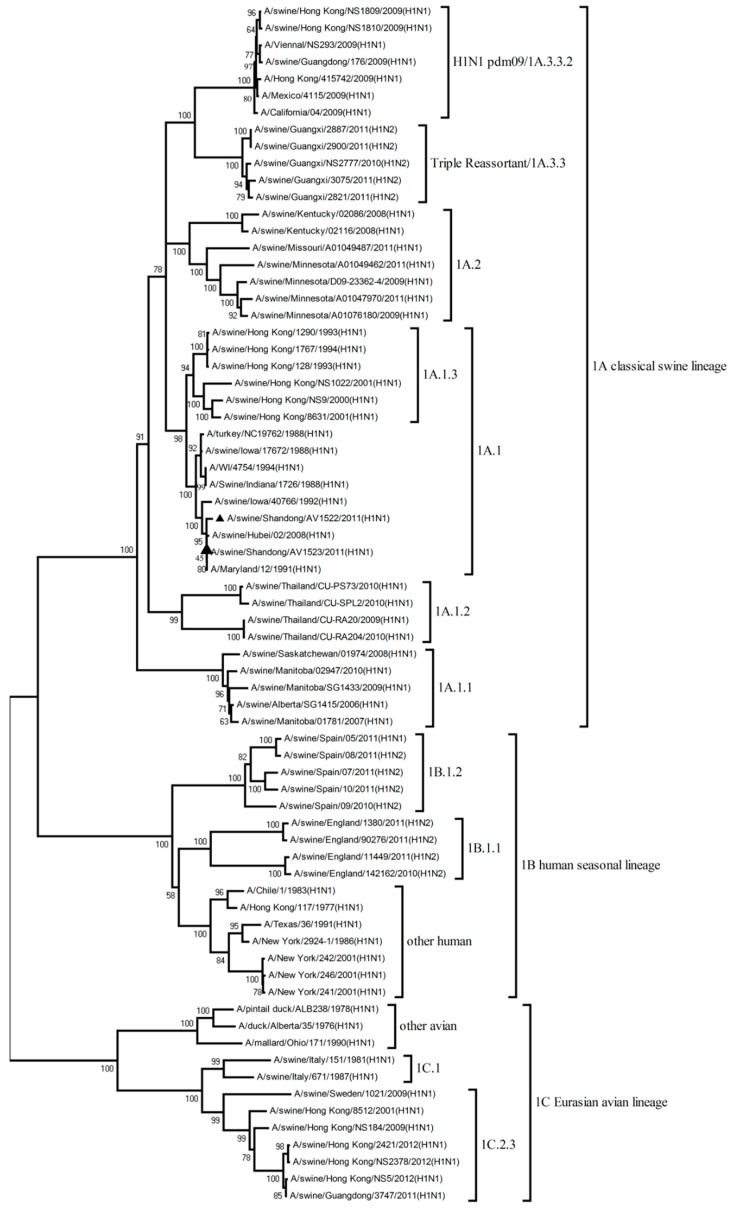
Phylogenetic analysis of the HA genes. The trees were constructed using the neighbor-joining method with the Maximum Composite Likelihood model and MEGA version 4.0 with 1000 bootstrap replicates. Our viruses were indicated by triangle marker “▲”.

The phylogenetic tree of the HA gene showed that the H1 viruses could be divided into three lineages: 1A classical swine lineage, 1B human seasonal lineage, and 1C Eurasian avian lineage. The HA genes of the AV1522 and AV1523 viruses were both clustered into 1A.1 sub-lineage of the 1A classical swine lineage. The other genes (NA, PB2, PB1, PA, NP, M, and NS) of the AV1522 virus all belonged to classical swine lineage. The NP gene of AV1523 virus fell into the H2N2-like sub-lineage of the human lineage. The other genes of the AV1523 virus belonged to the human lineage (Figure 1, Figure S1, Table 1, and Table S1).

Thus, by analyzing the phylogenetic tree and homology of the gene segments of the two viruses, the AV1522 virus seemed to be a descendant of classical swine influenza viruses and the AV1523 virus was a reassortant H1N1 influenza virus, containing genes from classical swine (HA) and human lineages (NA, PB2, PB1, PA, NP, M, and NS).

### 3.3. Molecular Characterization

To understand whether the signature amino acids associated with virulence, host adaptation, and drug resistance had changed, the deduced amino acid sequences of the two H1N1 viruses and the reference viruses from different lineages were analyzed.

**Figure 2:**
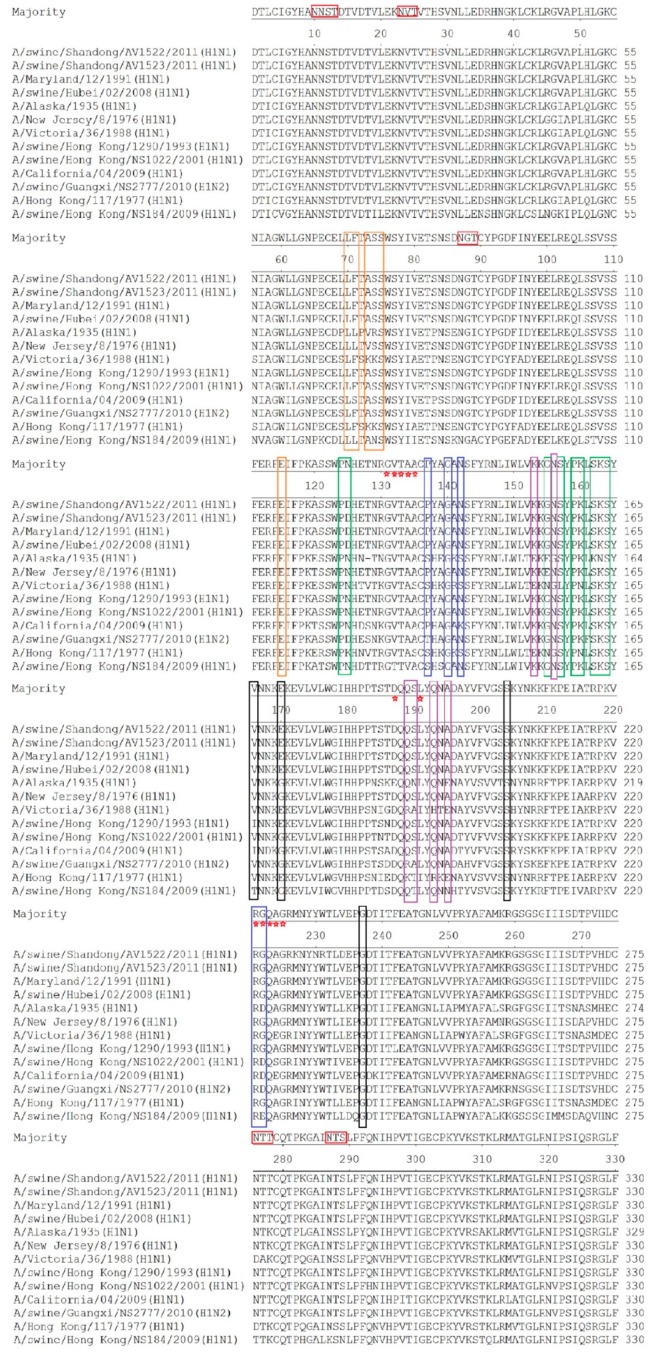
Molecular analysis of HA1 amino acid sequences of the two H1N1 swine influenza viruses and reference strains. Potential glycosylation sites were marked with red shade. Previously defined antigenic sites were indicated: Site Sa (green shade), site Sb (purple shade), site Ca1 (black shade), site Ca2 (blue shade), Cb (orange shade). The pentagram represents the receptor-binding sites. A/Maryland/12/1991(H1N1), A/swine/Hubei/02/2008(H1N1), A/swine/Hong Kong/1290/1993(H1N1), and A/swine/Hong Kong/NS1022/2001(H1N1) are all from the classical swine lineage. A/swine/Guangxi/NS2777/2010(H1N2) is from the triple reassortant sublineage of classical swine lineage. A/California/04/2009(H1N1) is from the pandemic H1N1/2009 sublineage of classical swine lineage. A/Alaska/1935(H1N1), A/New Jersey/1976(H1N1), A/Hong Kong/117/1977(H1N1) and A/Victoria/36/1988(H1N1) are all from the human lineage. A/swine/Hong Kong/NS184/2009(H1N1) is from the avian lineage.

The HA genes of the two H1N1 viruses both had an open reading frame of 1701 bp, encoding 566 amino acids. The AV1522 and the AV1523 viruses both contained the motif PSIQSR/GLF at the cleavage site between HA1 and HA2. No amino acid mutations or insertions happened at this position, and they met the characteristic of low pathogenic influenza viruses. The two H1N1 viruses both had six potential glycosylation sites (N-X-S/T) at positions 10 (NNS), 11 (NST), 23 (NVT), 87 (NGT), 276 (NTT), and 287 (NTS). Compared to the reference classical swine (CS) H1N1 influenza viruses, these glycosylation sites were relatively conservative (Figure 2).

The amino acid residues at positions 131–135, 187, 191, and 221–225 (H1 numbering used throughout) are well-established amino acid positions related to the receptor specificity of influenza viruses [20]. Amino acid residues at the receptor-binding pocket of HA1 (position Q223 and G225) retained configurations (2,6-NeuAcGal linkages) in the two H1N1 viruses of our study, predicting that they had an affinity for mammalian cell-surface receptors. The two H1N1 viruses and the reference viruses had the same amino acid residues at G131, T133, and R221. Though varying degrees of mutation at positions 132, 134, 135, 187, 191, and 222 occurred, the two H1N1 viruses had the same amino acids with the reference CS H1N1 influenza viruses isolated before 1993 (Figure 2).

The antigenic determinant domains (Sa, Sb, Ca1, Ca2, and Cb) were found to be involved in antibody binding [21,22]. The majority of amino acids in the antigenic determinant domains of the two H1N1 viruses of our study were similar to the reference CS H1N1 influenza viruses. In the antigenic site of Sa, mutation D125N was found in the two H1N1 viruses (Figure 2).

Mutations H274Y and N294S were not detected in the NA protein of the two H1N1 viruses, suggesting that the two H1N1 viruses were sensitive to neuraminidase inhibitors [23]. Single or multiple amino acid substitutions at positions L26F, V27A, A30T/V, S31N, G34E, and L38F in the transmembrane region of the M2 protein are the genetic basis for the resistance to amantadine and rimantadine [23,24]. L, A, G, and L were still maintained at positions 26, 30, 34, and 38, respectively. The AV1523 virus exhibited V27T and S31N amino acid substitutions in the M2 protein, and an V27I amino acid substitution was observed in the M2 protein of the AV1522 virus.

Amino acid residues in the polymerase, including PB2 mutations 199, 475, 567, 591, 627, 701, and 702, PB1 mutation 375, and PA mutations 55, 100, 382, and 552, are supposed to be host-specific markers of influenza viruses [25]. These above-mentioned amino acid residues in the two H1N1 viruses isolated in our study were identified. The results showed that amino acids residues at positions 199, 475, 567, 591, 627, and 701 in PB2, 375 in PB1, and 55, 100, and 382 in PA proteins were highly consistent with previous research. However, a R to K mutation was observed at position 702 of the PB2 protein in the AV1522 and AV1523 viruses. A S552T mutation was found in the PA protein of the AV1523 virus. The amino acid residues at positions 627 and 701 of the PB2 protein are also considered to be the predominant factor for the virulence of the influenza A viruses [26,27]. The PB2 E627K mutation was present in the AV1522 and AV1523 viruses. However, the amino acid residue at position 701 was still D in the two H1N1 viruses. The virulence of influenza viruses in humans is related to their resistance to the antiviral effects of cytokines, such as interferon (IFN), and the mutations P42S and D92E in the NS1 protein can increase resistance to IFN [28,29]. The P42S mutation was found in both of the two H1N1 viruses of our study, suggesting that these viruses may promote a greater resistance to these cytokines.

### 3.4. Pathogenicity of the Two H1N1 Viruses in BALB/c Mice

In general, mice serve as a useful animal model system for evaluating the virulence of influenza viruses to humans. In our study, SPF female BALB/c mice were inoculated with 10^6^EID_50_/50 μL of each virus and were monitored for 14 days for signs of illness, weight loss, and mortality. Three mice from each group were killed at 3 DPI, and a further three were killed at 5 DPI. Their brains, lungs, spleens, and kidneys were collected for virus titration in eggs.

**Figure 3:**
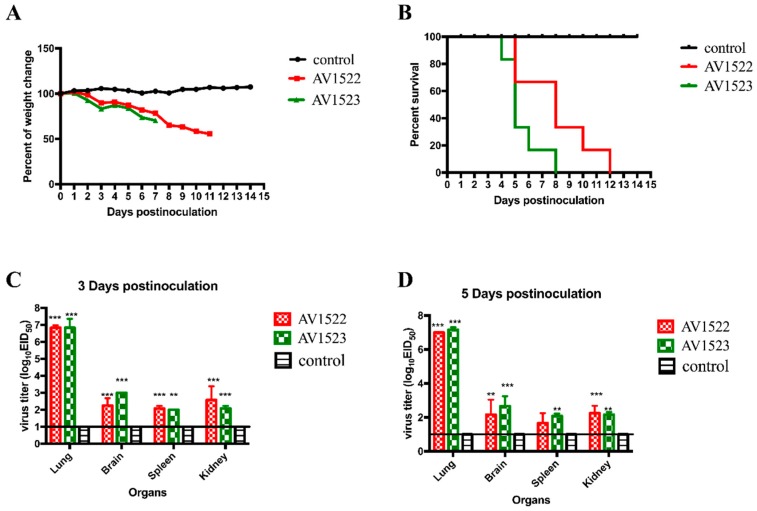
Weight change, lethality, and replication of the two H1N1 viruses in mice. (**A**) Weight change of BALB/c mice during the 14 days post-inoculation. Mice were inoculated intranasally with the doses of 10^6^EID_50_ of the H1N1 viruses. Mice inoculated with phosphate buffer saline (PBS) served as a control group. (**B**) Lethality of the AV1522 and AV1523 viruses in BALB/c mice. (**C**,**D**) Three six-week-old SPF BALB/c mice inoculated intranasally with 10^6^EID_50_ of each virus in a 50-μL volume were euthanized at 3 DPI and 5 DPI, and their organs were collected for virus titration in eggs. For statistical analysis, a value of 1.0 was assigned if the virus was not detected from the undiluted sample in three embryonated hen eggs. Data shown are the mean virus titers ± standard deviation in log_10_EID_50_/mL of tissue. Error bars indicate standard deviations.

The mice in the control group did not show any clinical symptoms and gained weight over the course of the observation period, and no virus was detected in the brains, lungs, spleens, or kidneys. The mice infected with the AV1522 virus displayed erect hair, chills, reduced appetite, and weight loss starting from 2 DPI, and all had died by 12 DPI. The AV1522 virus caused a systemic infection in the mice, replicated to the high titer of 6.8–7.0 log_10_EID_50_/mL in the lungs and to the mean titers of 2.3–2.2 log_10_EID_50_/mL, 2.0–1.7 log_10_EID_50_/mL, and 2.6–2.3 log_10_EID_50_/mL in the brains, spleens, and kidneys on 3 DPI and 5 DPI, respectively (Figure 3).

The mice infected with the AV1523 virus also showed signs of illness and a notable weight loss starting from 2 DPI. All of the mice infected with the AV1523 virus had died by 8 DPI. Viral replication of the AV1523 virus in lungs of the inoculated mice was also efficient, and the mean titers reached to 6.8–7.2 log_10_EID_50_/mL. The AV1523 virus could also be detected in the brains, spleens, and kidneys, and replicated to mean titers of 3.0–2.7 log_10_EID_50_/mL, 2.0–2.1 log_10_EID_50_/mL, and 2.1–2.2 log_10_EID_50_/mL, on 3 DPI and 5 DPI, respectively (Figure 3).

Overall, these data suggest that the two H1N1 viruses isolated from swine had a high pathogenicity to mice.

### 3.5. Histopathological Damage to Mouse Lungs and Brains Caused by AV1522 and AV1523 Viruses

**Figure 4:**
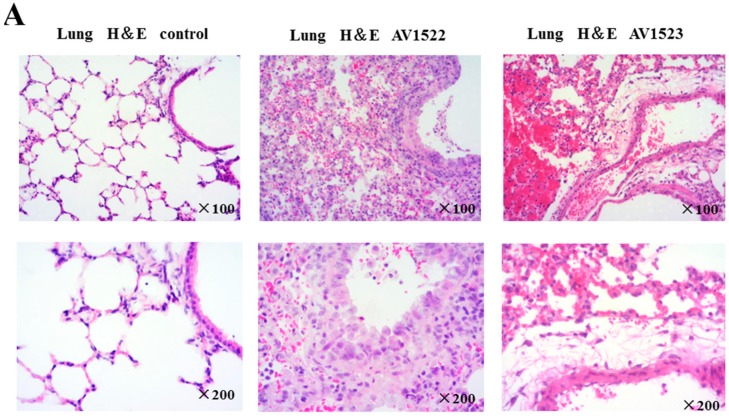

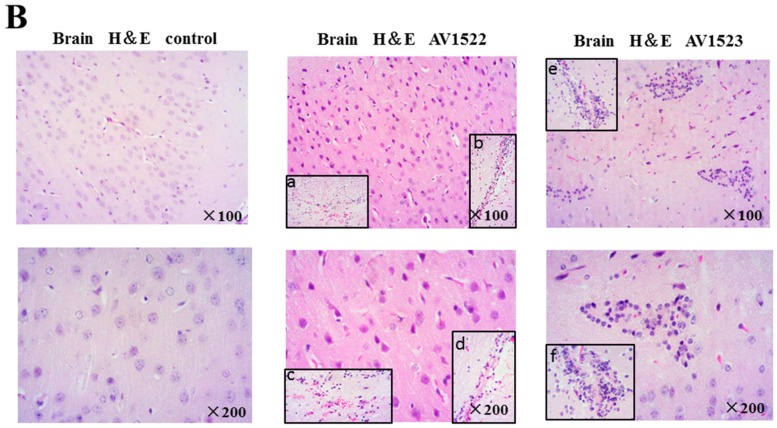
Histopathological examination of lung and brain tissues from mice infected with AV1522 virus or AV1523 virus. Representative images of hematoxylin-and-eosin staining tissues from mice inoculated with 10^6^EID_50_ of the indicated virus at 3 DPI and 5 DPI. (**A**) Moderate-to-marked necrotizing bronchiolitis and alveolitis, consisting of a mixed inflammatory infiltrate composed predominantly of neutrophils could be seen in lung tissues. Acute alveolar edema, hemorrhage, and congestion were also noted. (**B**) The brain tissues showed perivascular cuffing (b, d–f), multifocal microglial nodules (a, c), neuronophagia, and neuronal necrosis after infection with the AV1522 virus and AV1523 virus at 3 DPI and 5 DPI.

To correlate virulence with pathogenicity, lung and brain tissues from the inoculated mice were collected at 3 DPI and 5 DPI for pathological assessment. Mice inoculated with the AV1522 and AV1523 viruses both developed severe histopathological damage in the lungs and brains (Figure 4). Histopathological examinations of the lung tissue of mice infected with AV1522 and AV1523 viruses at 3 DPI revealed moderate-to-marked necrotizing bronchiolitis and alveolitis, consisting of a mixed inflammatory infiltrate composed predominantly of neutrophils. Focal areas of acute alveolar edema, hemorrhage, and congestion were also noted.

Analyses of hemotoxylin-and-eosin-stained brain sections showed that brain pathology caused by the AV1522 and AV1523 viruses at 3 DPI and 5 DPI was also severe and remarkable. Changes were already present in the brain by 3 DPI with the AV1522 virus and AV1523 virus, which rapidly progressed to perivascular cuffing, multifocal microglial nodules, neuronophagia, and neuronal necrosis in mice by 5 DPI.

Taken together, our data suggest that the AV1522 and AV1523 viruses caused severe disease and pathology in the mice model. The AV1522 and AV1523 viruses caused a spectrum of marked pneumonia and meningoencephalitis in accordance with the duration of infection, demonstrating a progression of respiratory disease and neurological disease over the course of infection that ultimately resulted in a lethal outcome for the majority of infected mice. Further histopathological evaluation of H1N1 swine viruses in mammalian models may help us to better understand the potential for severe disease in humans.

## 4. Discussion

Swine influenza was first recognized as a disease of pigs during the human pandemic in 1918. The virus was isolated in 1930, which is now known as classical swine H1N1 influenza virus (CS H1N1 virus) [30,31]. This CS H1N1 virus continued to circulate as the dominant influenza virus in the North American swine population until 1998. The CS H1N1 influenza might have occurred in pigs in cities along the Chinese coast during the 1918–1919 pandemic, and the virus was first isolated from Hong Kong of China in 1974 and from Mainland China in 1991. Influenza surveillance analysis conducted in China revealed that regular isolation of CS H1N1 viruses has been common in pigs since 1996 [10,32]. In our study, a CS H1N1 virus (A/swine/Shandong/AV1522/2011(H1N1)) was isolated from pigs with respiratory disease. Homology and phylogenetic analyses demonstrated that this virus had a close relationship with the CS H1N1 viruses isolated previously in North America and in China, which suggested that they might be descendants of the same ancestor.

Pigs are an important host in influenza A virus ecology, since they are susceptible to infection with influenza viruses from both birds and humans. Therefore, pigs have been known to serve as intermediate hosts for avian influenza viruses before humans, or as mixing vessels for the generation of genetically reassortant viruses. Avian/human, human/swine, and triple (human/avian/swine) reassortant influenza A viruses have been isolated from pigs worldwide. In this study, A/swine/Shandong/AV1523/2011(H1N1) was confirmed as a reassortant H1N1 influenza virus, containing genes from 1A classical swine lineage (HA) and human lineage (NA, PB2, PB1, PA, NP, M, and NS) [17,33,34]. Why did the reassortant event happen? Why were genes segments of the virus closely related to the human H1N1 and H2N2 viruses previously isolated from North America? Why did the human viral genes from many years ago still survive in the genome of the swine virus? One possible explanation may be that these human influenza viruses were introduced into pigs when they circulated in humans and persisted in pigs without antigenic drift. Pigs serve as reservoirs for older influenza viruses. Another possible explanation may be that pigs have a lifespan of approximately six months in China and no type of swine influenza vaccine has been used in pigs. Once the influenza viruses were introduced into pigs, these viruses might have appeared to have less immune selection pressure, and thus, the genes evolved more slowly than in humans and poultry. Furthermore, with rapid economic growth and increasing wealth, the number of imported pigs from North America and Europe increased rapidly in China, leading to enhanced opportunities for the spread of the swine influenza virus across the different continents [10]. Studies demonstrated that human and human-like H1N1 viruses were discovered in swine populations [35,36]. Therefore, we hypothesized that human H1N1 and H2N2 influenza viruses might have been introduced into pigs at the time they circulated in humans in North America and, subsequently, the reassortant event between the classical swine influenza and human influenza viruses occurred. As a result, accompanying the importation of pigs, the reassortant H1N1 influenza virus might be introduced into pigs in China. Previous studies found that the reassortant H1N1 viruses containing genes from human and classical swine influenza viruses had been isolated from pigs in China and other countries [37,38]. Therefore, to determine whether the reassortant event occurred before or after importation, further studies are required.

To evaluate the pathogenicity of the two H1N1 viruses in mammalian species, we inoculated BALB/c mice with 10^6^EID_50_/50μL of each virus and determined virus replication, mortality, and pathology. Previous studies showed that the H1N1 virus and its variant viruses isolated from different hosts and cases displayed a different pathogenicity in mice [39,40,41,42,43,44,45]. Those isolates showed effective replication in lungs but varied in their ability to cause morbidity. Memoli et al. found that A/Swine/Iowa/31 (Sw31) and the 1918 influenza viruses were uniformly lethal in mice at low dose, and produced severe lung pathology, whereas the recent human H1N1 virus (A/New York/312/2001) caused little disease in mice. Belser JA et al. showed that each of the 2009 H1N1 viruses studied replicated efficiently in the lungs of mice and possessed a high degree of infectivity but did not cause lethal disease or exhibit extrapulmonary virus spread. However, the triple reassortant OH/2 virus could result in greater weight loss and was lethal to mice at a dose of 10^5.8^ PFU. Zhu et al. demonstrated that the Eurasian avian-like influenza A(H1N1) virus was lethal in mice and had a substantially high virus titer in the nasal turbinates, trachea, and lungs and, occasionally, in the extrapulmonary organs (liver and kidney). Our results found that the two H1N1 viruses were highly pathogenic to mice and that they could both cause the death of all the mice during the experiments. More interestingly, the replication of the two H1N1 viruses were not restricted to respiratory tract tissues but could be systemic spread in the mice. The two H1N1 viruses in this study could not only be detected in the lungs, but also could replicate in the brains, spleens and kidneys of mice. Histopathological examinations of lung and brain tissues showed that the AV1522 and AV1523 viruses could cause severe disease and pathology in the mice. They caused a spectrum of marked pneumonia and meningoencephalitis in accordance with the duration of infection. A progression of respiratory disease and neurological disease over the course of infection of the two viruses resulted in a lethal outcome for the majority of infected mice. Therefore, the two H1N1 viruses in this study had a high pathogenicity to mice.

The pathogenicity of Influenza A viruses represents polygenic traits. Previous studies demonstrated that the evolution of swine influenza viruses progresses at a slower rate than in humans. Some classical swine viruses may retain the virulence factors of earlier classical swine H1N1 influenza viruses, such as the 1918 virus. Multiple studies linked variants in the HA segment of the influenza genome with enhanced mouse infectivity and virulence [46,47,48,49]. The HA genes of our two H1N1 viruses were both closely related to the early classical swine H1N1 influenza viruses which circulated in North America in the 1990s. The HA genes of two H1N1 viruses were both clustered into 1A.1 sub-lineage of the 1A classical swine lineage, which was derived from the 1918 human pandemic viruses [33]. Tumpey et al. found that the recombinant viruses possessing both the HA and NA genes of the 1918 influenza virus were highly lethal for mice. Whether these classical swine H1N1 influenza viruses and the A/Maryland/12/1991(H1N1) were all descendants of the 1918 virus, whether the HA gene played a role in the pathogenicity to mice, and whether the gene fragment provided by seasonal human influenza affected the pathogenicity of the virus to mice; each of these hypotheses require further studies. A few amino acid changes at critical sites of the HA gene (D125N), PB2 gene (E627K and R702K), and PA gene (S552T) might be responsible for pathogenic changes of the two H1N1 viruses in mice. This, too, requires further studies.

China has the largest human and pig populations in the world. From 1961 to 2016, China produced about 19 billion pigs, accounting for 46% of the world’s pigs. Approximately 130 million pigs have been imported into China [50]. (http://www.fao.org/faostat/en/#data/QA). Virologic surveillance revealed that CS H1N1, human-like H1N1, human-origin H3N2, European avian-like H1N1, northern American triple reassortant H1N2, and their reassortant variants were introduced into and cocirculated in pigs in China. The changes of pathogenicity of swine influenza viruses occur in mammals, emphasizing the importance of epidemiological investigations in pigs in China.

## Figures and Tables

**Table 1:** The influenza viruses in GenBank with highest nucleotide homology with AV1522 and AV1523 viruses when analyzing each gene fragment.

Virus	Gene	Virus with Highest Similarity	Homology (%)
A/swine/Shandong/AV1522/2011(H1N1)	HA	A/Maryland/12/1991(H1N1)	99.5
	NA	A/Maryland/12/1991(H1N1)	100
	PB2	A/Maryland/12/1991(H1N1)	99.8
	PB1	A/Maryland/12/1991(H1N1)	100
	PA	A/Maryland/12/1991(H1N1)	99.8
	NP	A/Maryland/12/1991(H1N1)	99.7
	M	A/Maryland/12/1991(H1N1)	99.9
	NS	A/Maryland/12/1991(H1N1)	100
A/swine/Shandong/AV1523/2011(H1N1)	HA	A/Maryland/12/1991(H1N1)	100
	NA	A/Alaska/1935(H1N1)	99.4
	PB2	A/Alaska/1935(H1N1)	100
	PB1	A/Alaska/1935(H1N1)	100
	PA	A/Victoria/36/1988(H1N1)	99.9
	NP	A/Ann Arbor/6/1960(H2N2)	99.7
	M	A/New Jersey/1976(H1N1)	99.8
	NS	A/Alaska/1935(H1N1)	99.8

## Supplementary Materials

The following are available online at https://www.mdpi.com/1999-4915/12/1/55/s1, Figure S1: Phylogenetic analysis of the NA(A), PB2 (B), PB1 (C), PA (D), NP (E), M (F), and NS (G)genes, Table S1: The nucleotide homology between the AV1522 virus and the A/swine/Hubei/02/2008(H1N1) virus, when analyzing each gene fragment.
