# Supplementary material for: Identification, Genetic Analysis, and Pathogenicity of Classical Swine H1N1 and Human-Swine Reassortant H1N1 Influenza Viruses from Pigs in China
Source: Viruses. 2020 Jan 2;12(1):55. doi: 10.3390/v12010055 (PMC7019673; doi:10.3390/v12010055)
Supplement: Supplementary file 1 [file viruses-12-00055-s001.pdf]

Identification, Genetic Analysis and Pathogenicity of Classical Swine H1N1 and Human-Swine Reassortant H1N1 Influenza Viruses from Pigs in China

Yafen Song[1], Yong Zhang[1], Bing Zhang [1], Ling Chen[1], Min Zhang[1], Jingwen Wang[1], Ying Jiang[1], Chenghuai Yang[1], Taozhen Jiang[1*]

[1] China Institute of Veterinary Drug Control, Beijing 100081, China

* Taozhen Jiang is the Corresponding author:

Dr. Taozhen Jiang, China Institute of Veterinary Drug Control, 8 Nandajie, Zhongguancun, Haidian District, Beijing, 100081, China.

Tel: (+86) 010-62103518; Fax: (+86) 010-61255380; E-mail: taozhen_jiang@163.com

Table S1. The nucleotide homology between the AV1522 virus and the A/swine/Hubei/02/2008(H1N1) virus, when analyzing each gene fragment.

virus	gene	virus with similarity	Homology (%)
A/swine/Shandong/AV1522/2011(H1N1)	HA	A/swine/Hubei/02/2008(H1N1)	99.5
	NA	A/swine/Hubei/02/2008(H1N1)	100
	PB2	A/swine/Hubei/02/2008(H1N1)	99.7
	PB1	A/swine/Hubei/02/2008(H1N1)	99.9
	PA	A/swine/Hubei/02/2008(H1N1)	99.6
	NP	A/swine/Hubei/02/2008(H1N1)	99.5
	M	A/swine/Hubei/02/2008(H1N1)	99.9
	NS	A/swine/Hubei/02/2008(H1N1)	100

Figure S1. Phylogenetic analysis of the NA(A), PB2 (B), PB1 (C), PA (D), NP (E), M (F), and NS (G)genes. The trees were constructed by using the neighbor-joining method with the Maximum Composite Likelihood model and MEGA version 4.0 with 1,000 bootstrap replicates. Our viruses were indicated by triangle marker "▲".

Figure S1A

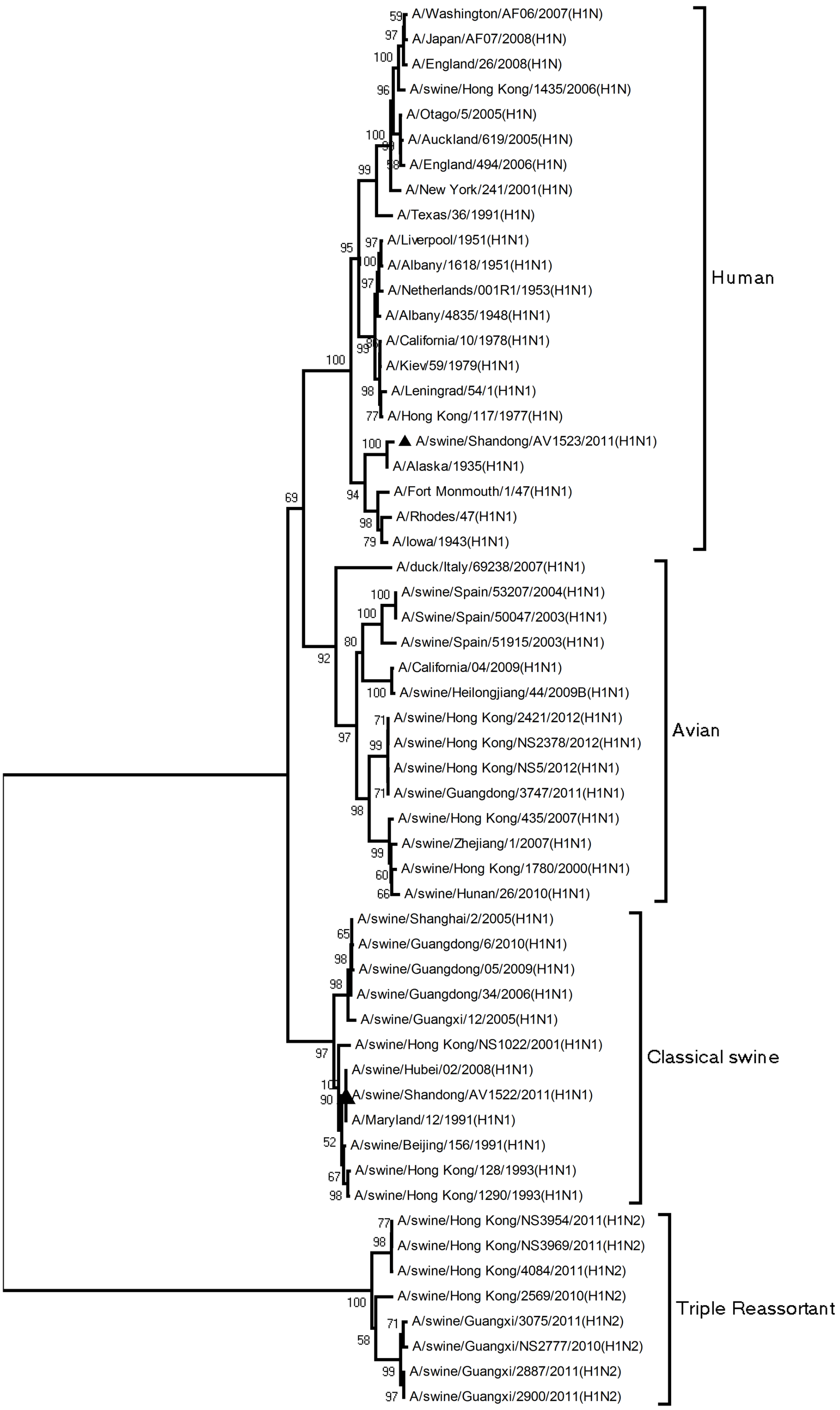

A/Washington/AF06/2007(H1N)
A/Japan/AF07/2008(H1N)
A/England/26/2008(H1N)
A/swine/Hong Kong/1435/2006(H1N)
A/Otago/5/2005(H1N)
A/Auckland/619/2005(H1N)
A/England/494/2006(H1N)
A/New York/241/2001(H1N)
A/Texas/36/1991(H1N)
A/Liverpool/1951(H1N1)
A/Albany/1618/1951(H1N1)
A/Netherlands/001R1/1953(H1N1)
A/Albany/4835/1948(H1N1)
A/California/10/1978(H1N1)
A/Kiev/59/1979(H1N1)
A/Leningrad/54/1(H1N1)
A/Hong Kong/117/1977(H1N)
A/swine/Shandong/AV1523/2011(H1N1)
A/Alaska/1935(H1N1)
A/Fort Monmouth/1/47(H1N1)
A/Rhodes/47(H1N1)
A/Iowa/1943(H1N1)
Human
A/duck/Italy/69238/2007(H1N1)
A/swine/Spain/53207/2004(H1N1)
A/Swine/Spain/50047/2003(H1N1)
A/swine/Spain/51915/2003(H1N1)
A/California/04/2009(H1N1)
A/swine/Heilongjiang/44/2009B(H1N1)
A/swine/Hong Kong/2421/2012(H1N1)
A/swine/Hong Kong/NS2378/2012(H1N1)
A/swine/Hong Kong/NS5/2012(H1N1)
A/swine/Guangdong/3747/2011(H1N1)
A/swine/Hong Kong/435/2007(H1N1)
A/swine/Zhejiang/1/2007(H1N1)
A/swine/Hong Kong/1780/2000(H1N1)
A/swine/Hunan/26/2010(H1N1)
Avian
A/swine/Shanghai/2/2005(H1N1)
A/swine/Guangdong/6/2010(H1N1)
A/swine/Guangdong/05/2009(H1N1)
A/swine/Guangdong/34/2006(H1N1)
A/swine/Guangxi/12/2005(H1N1)
A/swine/Hong Kong/NS1022/2001(H1N1)
A/swine/Hubei/02/2008(H1N1)
A/swine/Shandong/AV1522/2011(H1N1)
A/Maryland/12/1991(H1N1)
A/swine/Beijing/156/1991(H1N1)
A/swine/Hong Kong/128/1993(H1N1)
A/swine/Hong Kong/1290/1993(H1N1)
Classical swine
A/swine/Hong Kong/NS3954/2011(H1N2)
A/swine/Hong Kong/NS3969/2011(H1N2)
A/swine/Hong Kong/4084/2011(H1N2)
A/swine/Hong Kong/2569/2010(H1N2)
A/swine/Guangxi/3075/2011(H1N2)
A/swine/Guangxi/NS2777/2010(H1N2)
A/swine/Guangxi/2887/2011(H1N2)
A/swine/Guangxi/2900/2011(H1N2)
Triple Reassortant

Figure S1B

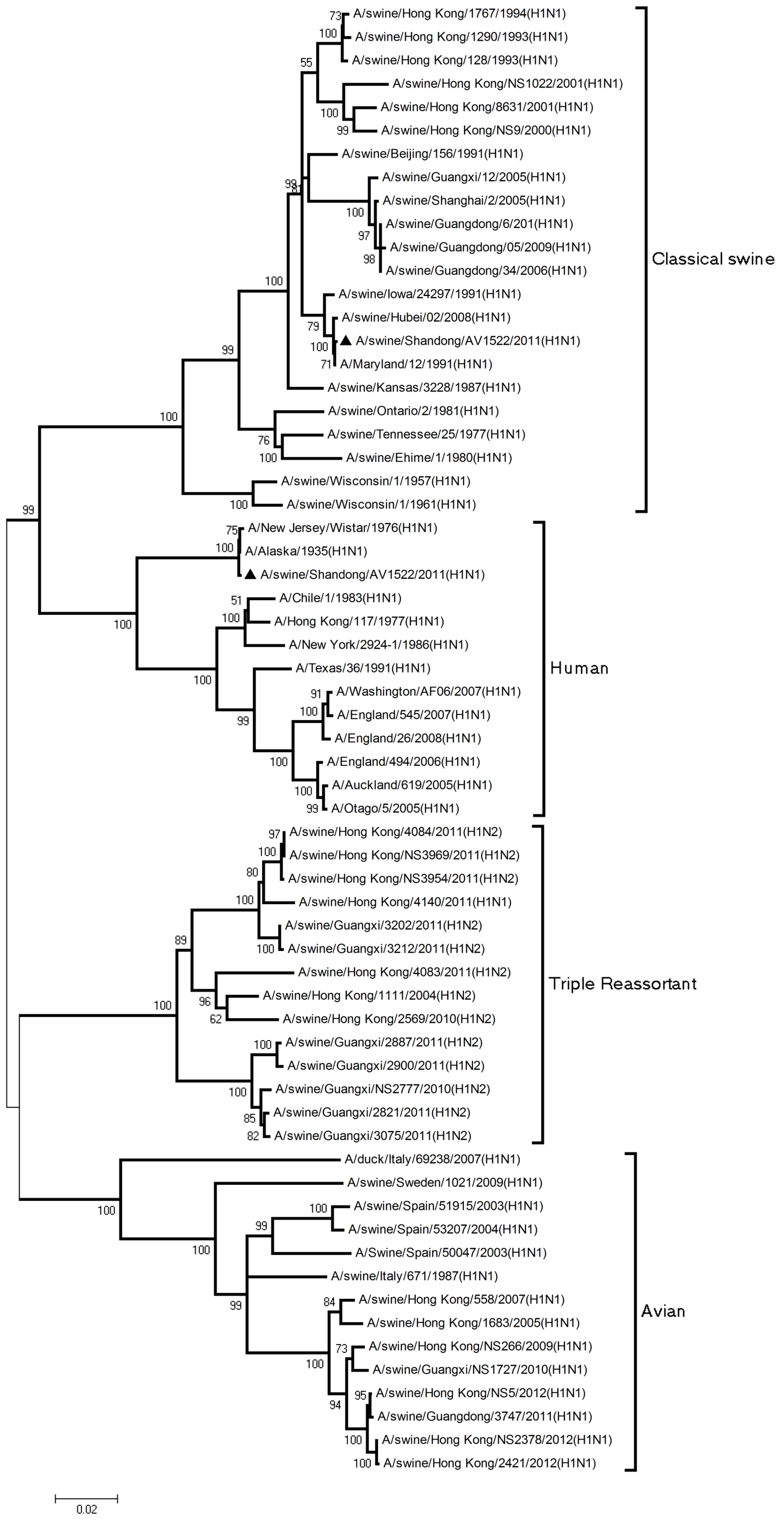
A/swine/Hong Kong/1767/1994(H1N1)
A/swine/Hong Kong/1290/1993(H1N1)
A/swine/Hong Kong/128/1993(H1N1)
A/swine/Hong Kong/NS1022/2001(H1N1)
A/swine/Hong Kong/8631/2001(H1N1)
A/swine/Hong Kong/NS9/2000(H1N1)
A/swine/Beijing/156/1991(H1N1)
A/swine/Guangxi/12/2005(H1N1)
A/swine/Shanghai/2/2005(H1N1)
A/swine/Guangdong/6/201(H1N1)
A/swine/Guangdong/05/2009(H1N1)
A/swine/Guangdong/34/2006(H1N1)
A/swine/Iowa/24297/1991(H1N1)
A/swine/Hubei/02/2008(H1N1)
A/swine/Shandong/AV1522/2011(H1N1)
A/Maryland/12/1991(H1N1)
A/swine/Kansas/3228/1987(H1N1)
A/swine/Ontario/2/1981(H1N1)
A/swine/Tennessee/25/1977(H1N1)
A/swine/Ehime/1/1980(H1N1)
A/swine/Wisconsin/1/1957(H1N1)
A/swine/Wisconsin/1/1961(H1N1)
Classical swine
A/New Jersey/Wistar/1976(H1N1)
A/Alaska/1935(H1N1)
A/swine/Shandong/AV1522/2011(H1N1)
A/Chile/1/1983(H1N1)
A/Hong Kong/117/1977(H1N1)
A/New York/2924-1/1986(H1N1)
A/Texas/36/1991(H1N1)
A/Washington/AF06/2007(H1N1)
A/England/545/2007(H1N1)
A/England/26/2008(H1N1)
A/England/494/2006(H1N1)
A/Auckland/619/2005(H1N1)
A/Otago/5/2005(H1N1)
Human
A/swine/Hong Kong/4084/2011(H1N2)
A/swine/Hong Kong/NS3969/2011(H1N2)
A/swine/Hong Kong/NS3954/2011(H1N2)
A/swine/Hong Kong/4140/2011(H1N1)
A/swine/Guangxi/3202/2011(H1N2)
A/swine/Guangxi/3212/2011(H1N2)
A/swine/Hong Kong/4083/2011(H1N2)
A/swine/Hong Kong/1111/2004(H1N2)
A/swine/Hong Kong/2569/2010(H1N2)
A/swine/Guangxi/2887/2011(H1N2)
A/swine/Guangxi/2900/2011(H1N2)
A/swine/Guangxi/NS2777/2010(H1N2)
A/swine/Guangxi/2821/2011(H1N2)
A/swine/Guangxi/3075/2011(H1N2)
Triple Reassortant
A/duck/Italy/69238/2007(H1N1)
A/swine/Sweden/1021/2009(H1N1)
A/swine/Spain/51915/2003(H1N1)
A/swine/Spain/53207/2004(H1N1)
A/Swine/Spain/50047/2003(H1N1)
A/swine/Italy/671/1987(H1N1)
A/swine/Hong Kong/558/2007(H1N1)
A/swine/Hong Kong/1683/2005(H1N1)
A/swine/Hong Kong/NS266/2009(H1N1)
A/swine/Guangxi/NS1727/2010(H1N1)
A/swine/Hong Kong/NS5/2012(H1N1)
A/swine/Guangdong/3747/2011(H1N1)
A/swine/Hong Kong/NS2378/2012(H1N1)
A/swine/Hong Kong/2421/2012(H1N1)
Avian
0.02

Figure S1C

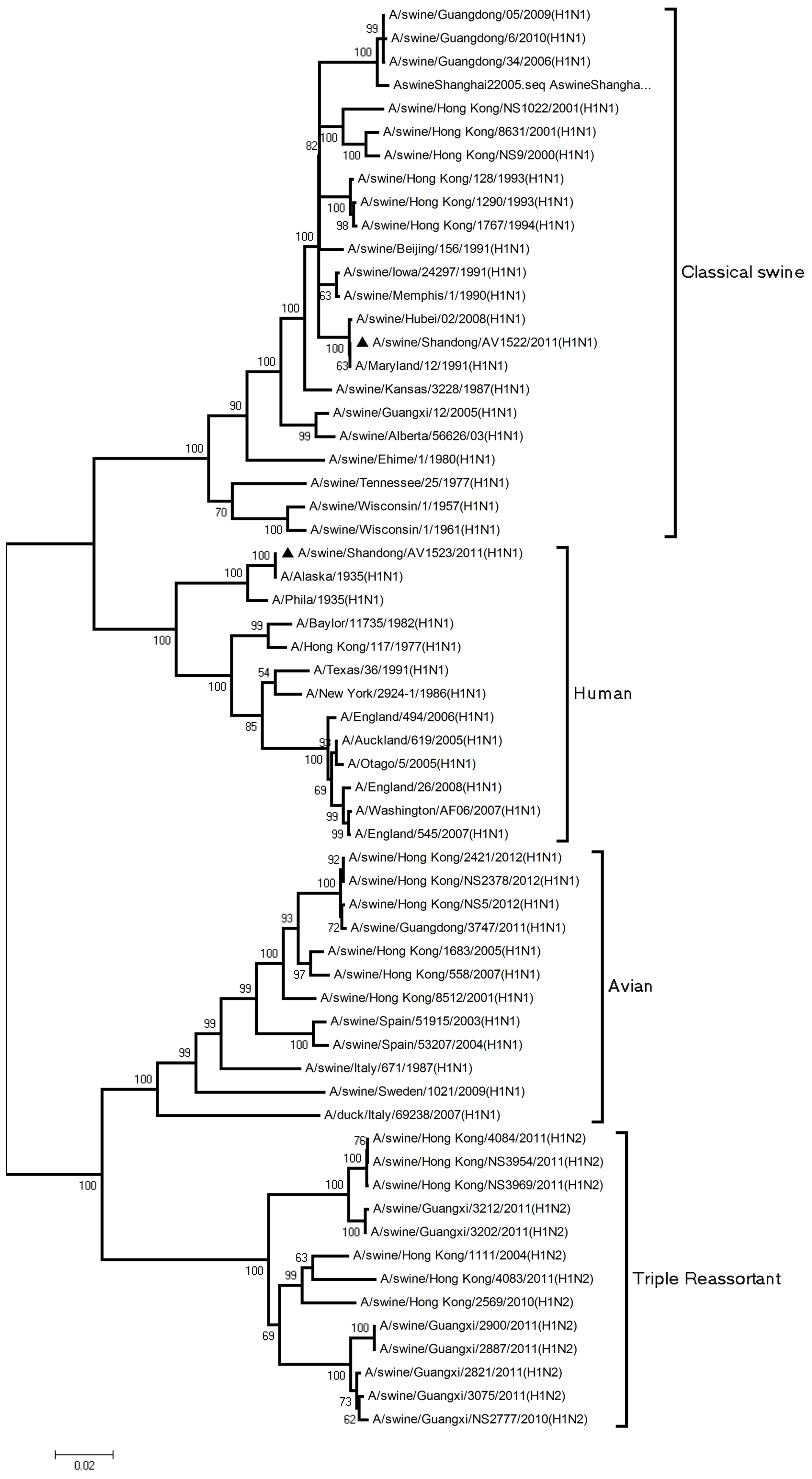
A/swine/Guangdong/05/2009(H1N1)
A/swine/Guangdong/6/2010(H1N1)
A/swine/Guangdong/34/2006(H1N1)
AswineShanghai22005.seq AswineShangha...
A/swine/Hong Kong/NS1022/2001(H1N1)
A/swine/Hong Kong/8631/2001(H1N1)
A/swine/Hong Kong/NS9/2000(H1N1)
A/swine/Hong Kong/128/1993(H1N1)
A/swine/Hong Kong/1290/1993(H1N1)
A/swine/Hong Kong/1767/1994(H1N1)
A/swine/Beijing/156/1991(H1N1)
A/swine/Iowa/24297/1991(H1N1)
A/swine/Memphis/1/1990(H1N1)
A/swine/Hubei/02/2008(H1N1)
A/swine/Shandong/AV1522/2011(H1N1)
A/Maryland/12/1991(H1N1)
A/swine/Kansas/3228/1987(H1N1)
A/swine/Guangxi/12/2005(H1N1)
A/swine/Alberta/56626/03(H1N1)
A/swine/Ehime/1/1980(H1N1)
A/swine/Tennessee/25/1977(H1N1)
A/swine/Wisconsin/1/1957(H1N1)
A/swine/Wisconsin/1/1961(H1N1)
Classical swine
A/swine/Shandong/AV1523/2011(H1N1)
A/Alaska/1935(H1N1)
A/Phila/1935(H1N1)
A/Baylor/11735/1982(H1N1)
A/Hong Kong/117/1977(H1N1)
A/Texas/36/1991(H1N1)
A/New York/2924-1/1986(H1N1)
A/England/494/2006(H1N1)
A/Auckland/619/2005(H1N1)
A/Otago/5/2005(H1N1)
A/England/26/2008(H1N1)
A/Washington/AF06/2007(H1N1)
A/England/545/2007(H1N1)
Human
A/swine/Hong Kong/2421/2012(H1N1)
A/swine/Hong Kong/NS2378/2012(H1N1)
A/swine/Hong Kong/NS5/2012(H1N1)
A/swine/Guangdong/3747/2011(H1N1)
A/swine/Hong Kong/1683/2005(H1N1)
A/swine/Hong Kong/558/2007(H1N1)
A/swine/Hong Kong/8512/2001(H1N1)
A/swine/Spain/51915/2003(H1N1)
A/swine/Spain/53207/2004(H1N1)
A/swine/Italy/671/1987(H1N1)
A/swine/Sweden/1021/2009(H1N1)
A/duck/Italy/69238/2007(H1N1)
Avian
A/swine/Hong Kong/4084/2011(H1N2)
A/swine/Hong Kong/NS3954/2011(H1N2)
A/swine/Hong Kong/NS3969/2011(H1N2)
A/swine/Guangxi/3212/2011(H1N2)
A/swine/Guangxi/3202/2011(H1N2)
A/swine/Hong Kong/1111/2004(H1N2)
A/swine/Hong Kong/4083/2011(H1N2)
A/swine/Hong Kong/2569/2010(H1N2)
A/swine/Guangxi/2900/2011(H1N2)
A/swine/Guangxi/2887/2011(H1N2)
A/swine/Guangxi/2821/2011(H1N2)
A/swine/Guangxi/3075/2011(H1N2)
A/swine/Guangxi/NS2777/2010(H1N2)
Triple Reassortant
0.02

Figure S1D

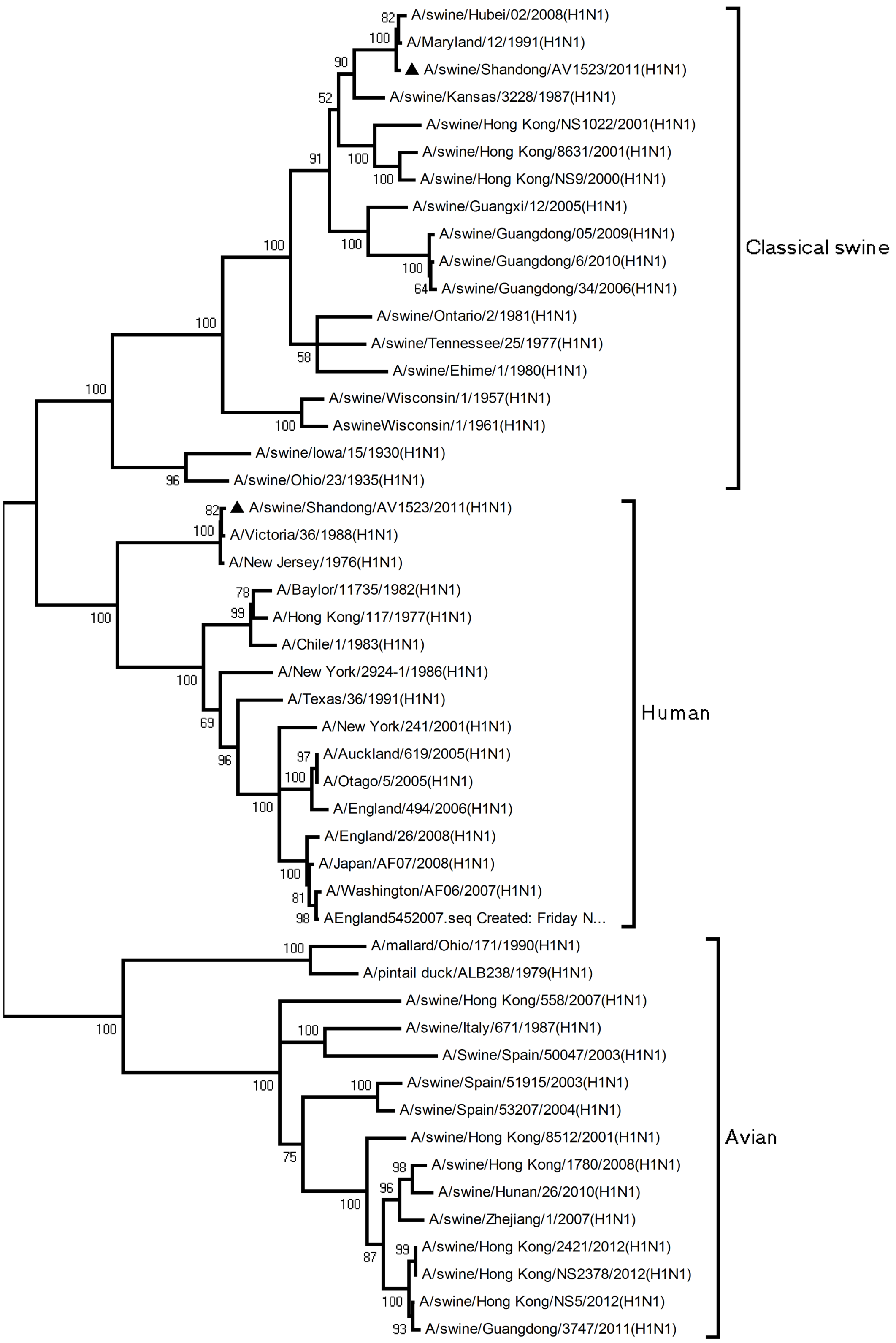
A/swine/Hubei/02/2008(H1N1)
A/Maryland/12/1991(H1N1)
A/swine/Shandong/AV1523/2011(H1N1)
A/swine/Kansas/3228/1987(H1N1)
A/swine/Hong Kong/NS1022/2001(H1N1)
A/swine/Hong Kong/8631/2001(H1N1)
A/swine/Hong Kong/NS9/2000(H1N1)
A/swine/Guangxi/12/2005(H1N1)
A/swine/Guangdong/05/2009(H1N1)
A/swine/Guangdong/6/2010(H1N1)
A/swine/Guangdong/34/2006(H1N1)
A/swine/Ontario/2/1981(H1N1)
A/swine/Tennessee/25/1977(H1N1)
A/swine/Ehime/1/1980(H1N1)
A/swine/Wisconsin/1/1957(H1N1)
AswineWisconsin/1/1961(H1N1)
A/swine/Iowa/15/1930(H1N1)
A/swine/Ohio/23/1935(H1N1)
Classical swine
A/swine/Shandong/AV1523/2011(H1N1)
A/Victoria/36/1988(H1N1)
A/New Jersey/1976(H1N1)
A/Baylor/11735/1982(H1N1)
A/Hong Kong/117/1977(H1N1)
A/Chile/1/1983(H1N1)
A/New York/2924-1/1986(H1N1)
A/Texas/36/1991(H1N1)
A/New York/241/2001(H1N1)
A/Auckland/619/2005(H1N1)
A/Otago/5/2005(H1N1)
A/England/494/2006(H1N1)
A/England/26/2008(H1N1)
A/Japan/AF07/2008(H1N1)
A/Washington/AF06/2007(H1N1)
AEngland5452007.seq Created: Friday N...
Human
A/mallard/Ohio/171/1990(H1N1)
A/pintail duck/ALB238/1979(H1N1)
A/swine/Hong Kong/558/2007(H1N1)
A/swine/Italy/671/1987(H1N1)
A/Swine/Spain/50047/2003(H1N1)
A/swine/Spain/51915/2003(H1N1)
A/swine/Spain/53207/2004(H1N1)
A/swine/Hong Kong/8512/2001(H1N1)
A/swine/Hong Kong/1780/2008(H1N1)
A/swine/Hunan/26/2010(H1N1)
A/swine/Zhejiang/1/2007(H1N1)
A/swine/Hong Kong/2421/2012(H1N1)
A/swine/Hong Kong/NS2378/2012(H1N1)
A/swine/Hong Kong/NS5/2012(H1N1)
A/swine/Guangdong/3747/2011(H1N1)
Avian

Figure S1E

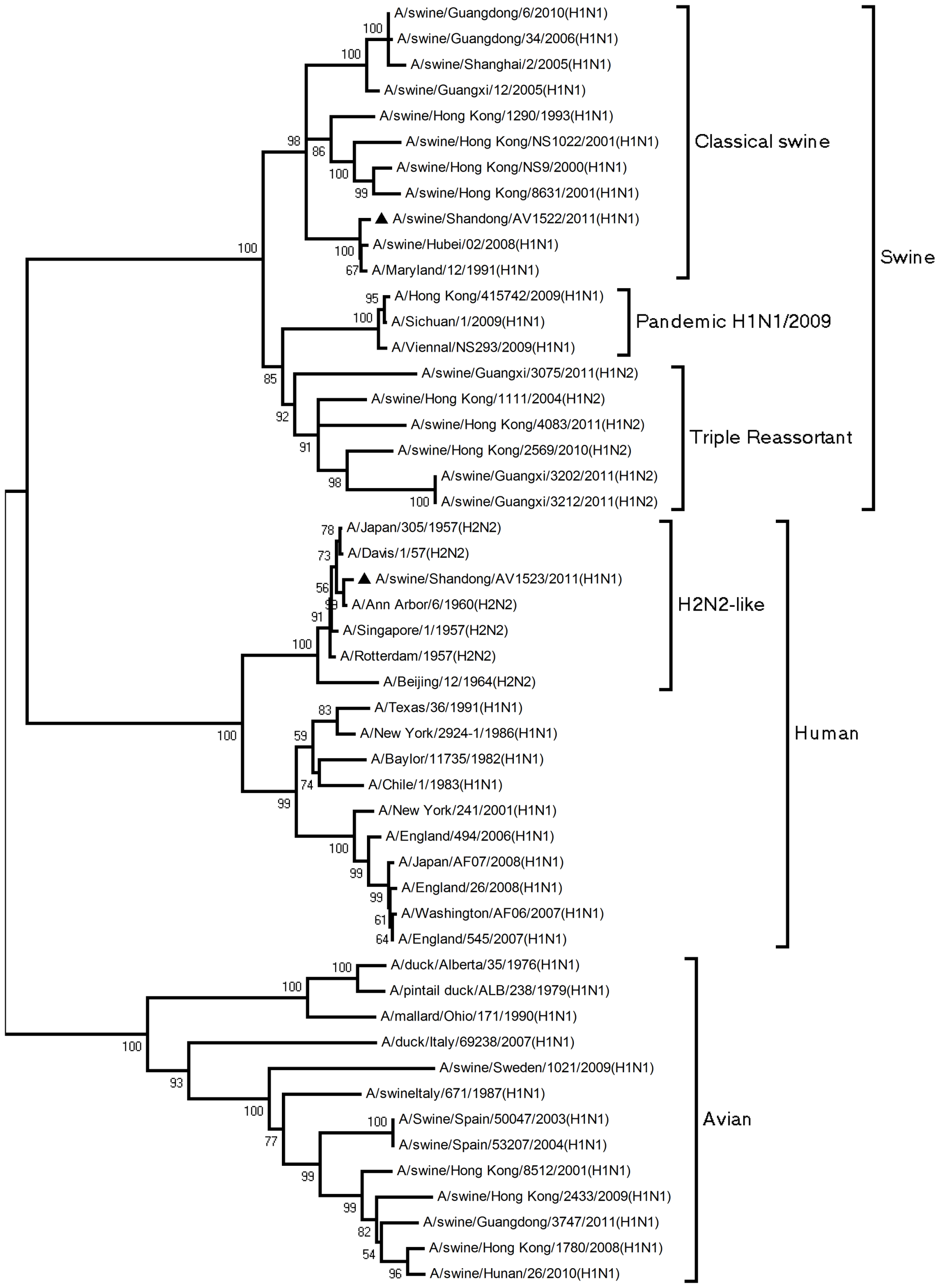
A/swine/Guangdong/6/2010(H1N1)
A/swine/Guangdong/34/2006(H1N1)
A/swine/Shanghai/2/2005(H1N1)
A/swine/Guangxi/12/2005(H1N1)
A/swine/Hong Kong/1290/1993(H1N1)
A/swine/Hong Kong/NS1022/2001(H1N1)
A/swine/Hong Kong/NS9/2000(H1N1)
A/swine/Hong Kong/8631/2001(H1N1)
▲ A/swine/Shandong/AV1522/2011(H1N1)
A/swine/Hubei/02/2008(H1N1)
A/Maryland/12/1991(H1N1)
A/Hong Kong/415742/2009(H1N1)
A/Sichuan/1/2009(H1N1)
A/Viennal/NS293/2009(H1N1)
A/swine/Guangxi/3075/2011(H1N2)
A/swine/Hong Kong/1111/2004(H1N2)
A/swine/Hong Kong/4083/2011(H1N2)
A/swine/Hong Kong/2569/2010(H1N2)
A/swine/Guangxi/3202/2011(H1N2)
A/swine/Guangxi/3212/2011(H1N2)
A/Japan/305/1957(H2N2)
A/Davis/1/57(H2N2)
▲ A/swine/Shandong/AV1523/2011(H1N1)
A/Ann Arbor/6/1960(H2N2)
A/Singapore/1/1957(H2N2)
A/Rotterdam/1957(H2N2)
A/Beijing/12/1964(H2N2)
A/Texas/36/1991(H1N1)
A/New York/2924-1/1986(H1N1)
A/Baylor/11735/1982(H1N1)
A/Chile/1/1983(H1N1)
A/New York/241/2001(H1N1)
A/England/494/2006(H1N1)
A/Japan/AF07/2008(H1N1)
A/England/26/2008(H1N1)
A/Washington/AF06/2007(H1N1)
A/England/545/2007(H1N1)
A/duck/Alberta/35/1976(H1N1)
A/pintail duck/ALB/238/1979(H1N1)
A/mallard/Ohio/171/1990(H1N1)
A/duck/Italy/69238/2007(H1N1)
A/swine/Sweden/1021/2009(H1N1)
A/swineItaly/671/1987(H1N1)
A/Swine/Spain/50047/2003(H1N1)
A/swine/Spain/53207/2004(H1N1)
A/swine/Hong Kong/8512/2001(H1N1)
A/swine/Hong Kong/2433/2009(H1N1)
A/swine/Guangdong/3747/2011(H1N1)
A/swine/Hong Kong/1780/2008(H1N1)
A/swine/Hunan/26/2010(H1N1)
Classical swine
Pandemic H1N1/2009
Triple Reassortant
Swine
H2N2-like
Human
Avian

Figure S1F

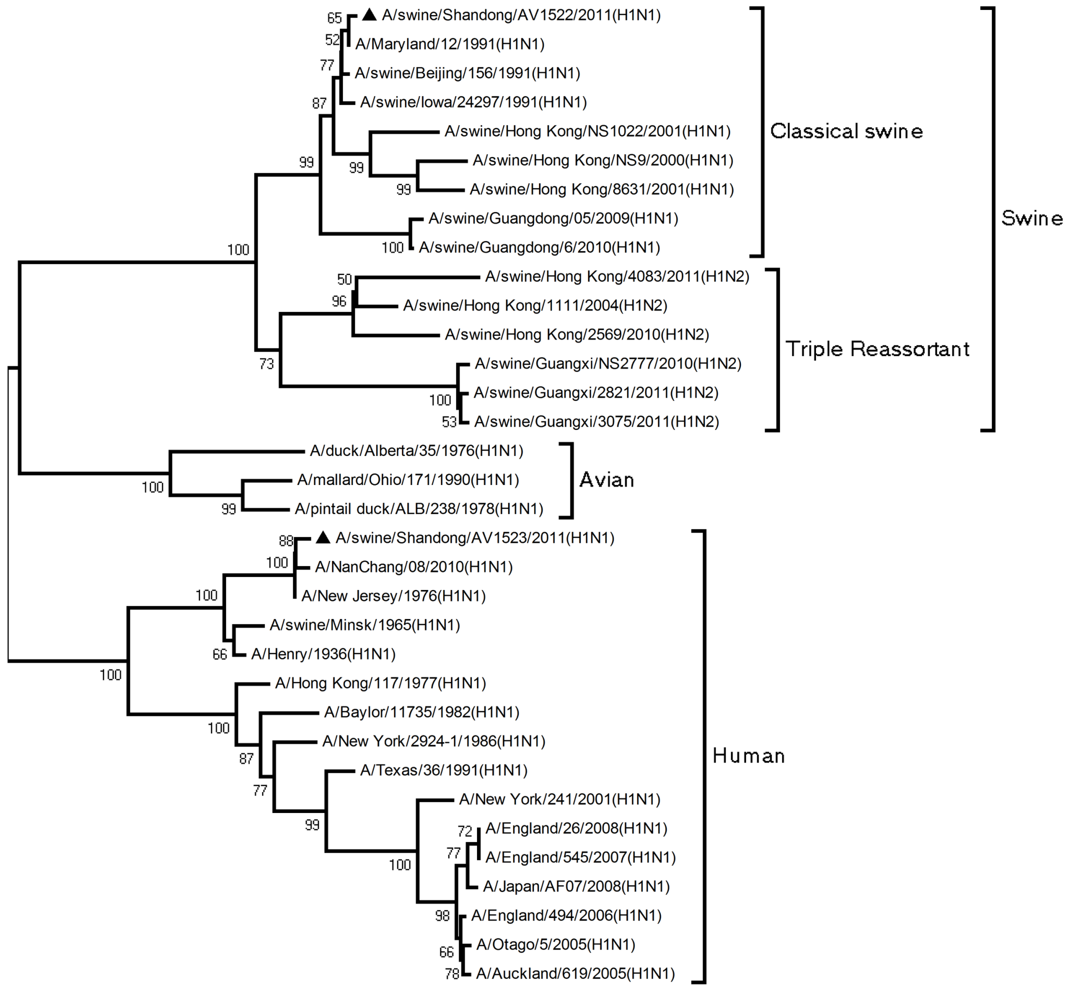
A/swine/Shandong/AV1522/2011(H1N1)
A/Maryland/12/1991(H1N1)
A/swine/Beijing/156/1991(H1N1)
A/swine/Iowa/24297/1991(H1N1)
A/swine/Hong Kong/NS1022/2001(H1N1)
A/swine/Hong Kong/NS9/2000(H1N1)
A/swine/Hong Kong/8631/2001(H1N1)
A/swine/Guangdong/05/2009(H1N1)
A/swine/Guangdong/6/2010(H1N1)
A/swine/Hong Kong/4083/2011(H1N2)
A/swine/Hong Kong/1111/2004(H1N2)
A/swine/Hong Kong/2569/2010(H1N2)
A/swine/Guangxi/NS2777/2010(H1N2)
A/swine/Guangxi/2821/2011(H1N2)
A/swine/Guangxi/3075/2011(H1N2)
A/duck/Alberta/35/1976(H1N1)
A/mallard/Ohio/171/1990(H1N1)
A/pintail duck/ALB/238/1978(H1N1)
A/swine/Shandong/AV1523/2011(H1N1)
A/NanChang/08/2010(H1N1)
A/New Jersey/1976(H1N1)
A/swine/Minsk/1965(H1N1)
A/Henry/1936(H1N1)
A/Hong Kong/117/1977(H1N1)
A/Baylor/11735/1982(H1N1)
A/New York/2924-1/1986(H1N1)
A/Texas/36/1991(H1N1)
A/New York/241/2001(H1N1)
A/England/26/2008(H1N1)
A/England/545/2007(H1N1)
A/Japan/AF07/2008(H1N1)
A/England/494/2006(H1N1)
A/Otago/5/2005(H1N1)
A/Auckland/619/2005(H1N1)
Classical swine
Triple Reassortant
Swine
Avian
Human

Figure S1G

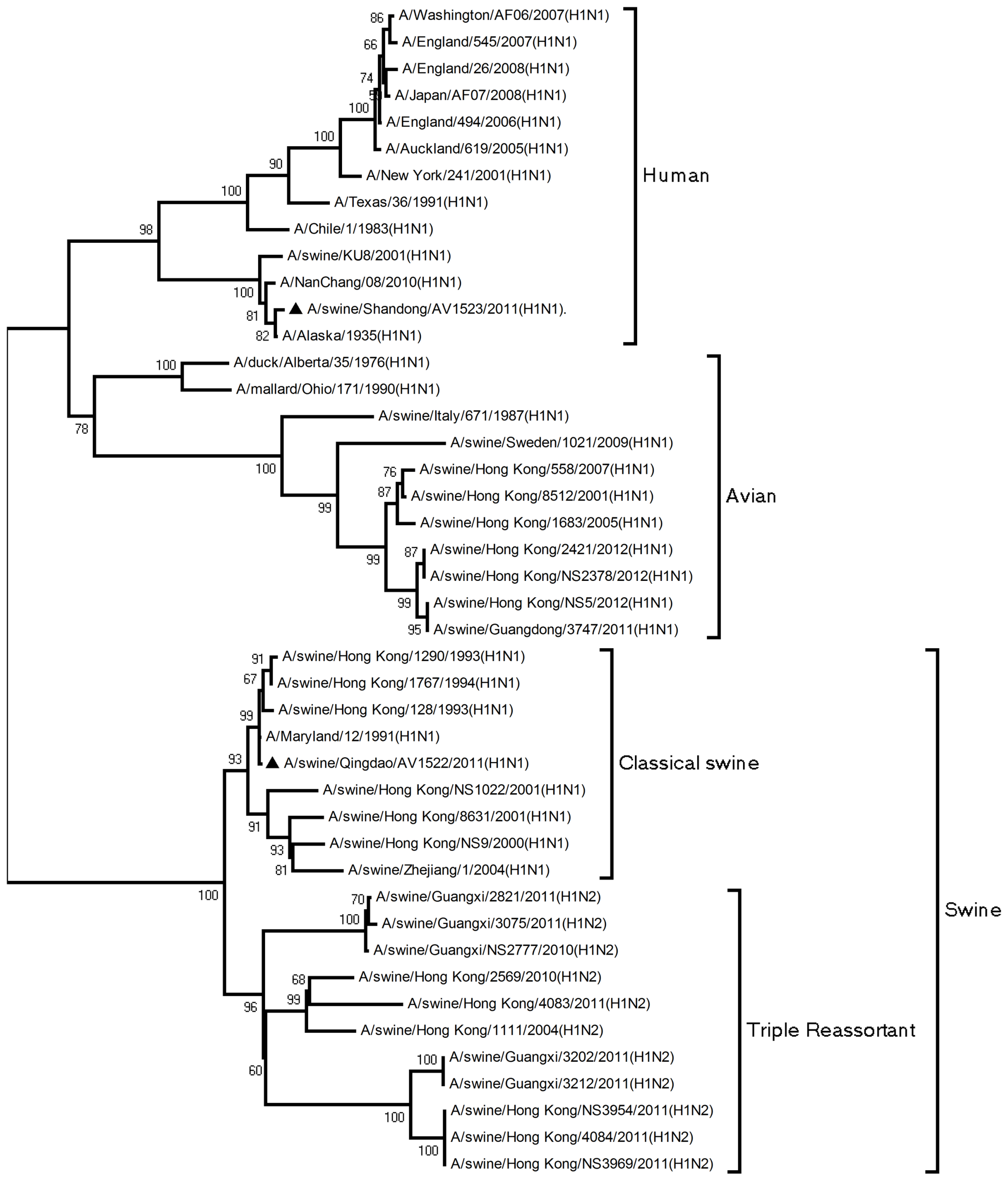

A/Washington/AF06/2007(H1N1)
A/England/545/2007(H1N1)
A/England/26/2008(H1N1)
A/Japan/AF07/2008(H1N1)
A/England/494/2006(H1N1)
A/Auckland/619/2005(H1N1)
A/New York/241/2001(H1N1)
A/Texas/36/1991(H1N1)
A/Chile/1/1983(H1N1)
A/swine/KU8/2001(H1N1)
A/NanChang/08/2010(H1N1)
▲ A/swine/Shandong/AV1523/2011(H1N1).
A/Alaska/1935(H1N1)
Human
A/duck/Alberta/35/1976(H1N1)
A/mallard/Ohio/171/1990(H1N1)
A/swine/Italy/671/1987(H1N1)
A/swine/Sweden/1021/2009(H1N1)
A/swine/Hong Kong/558/2007(H1N1)
A/swine/Hong Kong/8512/2001(H1N1)
A/swine/Hong Kong/1683/2005(H1N1)
A/swine/Hong Kong/2421/2012(H1N1)
A/swine/Hong Kong/NS2378/2012(H1N1)
A/swine/Hong Kong/NS5/2012(H1N1)
A/swine/Guangdong/3747/2011(H1N1)
Avian
A/swine/Hong Kong/1290/1993(H1N1)
A/swine/Hong Kong/1767/1994(H1N1)
A/swine/Hong Kong/128/1993(H1N1)
A/Maryland/12/1991(H1N1)
▲ A/swine/Qingdao/AV1522/2011(H1N1)
A/swine/Hong Kong/NS1022/2001(H1N1)
A/swine/Hong Kong/8631/2001(H1N1)
A/swine/Hong Kong/NS9/2000(H1N1)
A/swine/Zhejiang/1/2004(H1N1)
Classical swine
A/swine/Guangxi/2821/2011(H1N2)
A/swine/Guangxi/3075/2011(H1N2)
A/swine/Guangxi/NS2777/2010(H1N2)
A/swine/Hong Kong/2569/2010(H1N2)
A/swine/Hong Kong/4083/2011(H1N2)
A/swine/Hong Kong/1111/2004(H1N2)
A/swine/Guangxi/3202/2011(H1N2)
A/swine/Guangxi/3212/2011(H1N2)
A/swine/Hong Kong/NS3954/2011(H1N2)
A/swine/Hong Kong/4084/2011(H1N2)
A/swine/Hong Kong/NS3969/2011(H1N2)
Triple Reassortant
Swine
